# 1775. Interns and Residents’ Knowledge and Perspective on Appropriate Use of Antibiotics for Urinary Tract Infections and Skin and Soft Tissue Infections in the Hospital Setting

**DOI:** 10.1093/ofid/ofac492.1405

**Published:** 2022-12-15

**Authors:** Gabriel Motoa, Joseph Borick, Monisha Bhatia, Lauren Bjork, Paola Lichtenberger

**Affiliations:** University of Miami/Jackson Memorial Hospital, Miami, Florida; Jackson Memorial Hospital, Miami, Florida; Miami VA Medical Center, Miami, Florida; Miami VA Medical Center, Miami, Florida; Infectious Disease Division. University of Miami, Miller School of Medicine., Miami, Florida

## Abstract

**Background:**

Antibiotic resistance is recognized as a significant threat to public health worldwide. The rate of inappropriate use of antibiotics in hospitals has been reported as high as 50%. Exposure to suboptimal antibiotic therapy is associated with higher risk of infections with multidrug-resistant organisms and worse clinical outcomes. In academic hospitals, interns and residents are frontline worker who diagnose and treat infectious diseases (ID). The treatment of urinary tract infections (UTIs) and skin and soft tissue infections (SSTIs) is a common clinical scenario managed by these trainees.

**Methods:**

A structured, anonymous questionnaire was sent via institutional email to internal medicine interns and residents rotating in three academic hospitals in Miami, FL. The 23-item questionnaire included knowledge questions on rational use of antibiotics and duration of therapy for complicated and uncomplicated UTIs, SSTIs, correct renal dosing of frequently used antibiotics and antivirals, and perception of confidence in antibiotic prescription. Data were entered to Microsoft Excel spreadsheet, then analyzed using JMP Pro software.

**Results:**

A total of 36 preliminary and categorical interns, and 38 second-and third-year residents completed the initial questionnaire. The mean correct knowledge score on the treatment of UTIs was 52.8% (19/36) for interns and 76.3% (29/38) for residents, respectively. For SSTIs, the scores were 38.9% (14/36) and 65.8% (25/38); and for correct renal dosing of antibiotics, the scores were 47.2% (17/36) vs. 73.7% (28/38). Most of the interns (58.3%, 21/36) did not feel confident prescribing antibiotics for these conditions compared with 18.4% (7/38) of the residents.

**Conclusion:**

In this cross-sectional study, interns had lower correct knowledge scores and confidence when prescribing antibiotics for UTIs and SSTIs compared to residents. There is room for improvement in advanced years of training. Further studies are necessary to assess the impact of implementing educational strategies to promote the appropriate use of antibiotics. We plan to address this through a pre-/post implementation We will compare antibiotic prescribing patterns and patient outcomes for these indications at one of our three clinical training sites.

**Disclosures:**

**All Authors**: No reported disclosures.

